# Transplantation of bone marrow-derived mesenchymal stem cells with silencing of microRNA-138 relieves pelvic organ prolapse through the FBLN5/IL-1β/elastin pathway

**DOI:** 10.18632/aging.202465

**Published:** 2021-01-16

**Authors:** Bing Zhao, Qing Sun, Yazhou Fan, Xinming Hu, Linyu Li, Junmin Wang, Shihong Cui

**Affiliations:** 1 Department of Gynaecology and Obstetrics, The Third Affiliated Hospital of Zhengzhou University, Zhengzhou 450052, Henan Province, PR China; 2 Department of Scientific Research, Xinxiang Medical University Sanquan Medical College, Xinxiang 453003, Henan Province, PR China; 3 Department of Anatomy, College of Basic Medical Sciences, Zhengzhou University, Zhengzhou 450000, Henan Province, PR China

**Keywords:** pelvic organ prolapse, bone marrow-derived mesenchymal stem cells, microRNA-138, FBLN5, IL-1β

## Abstract

Nondegradable transvaginal polypropylene meshes for treating pelvic organ prolapse (POP) are now generally unavailable or banned due to serious adverse events. New tissue engineering approaches combine degradable scaffolds with mesenchymal stem/stromal cells from human endometrium (eMSC). In this study, we investigate effect of microRNA-138 (miR-138) regulation on bone marrow-derived mesenchymal stem cells (BMSCs) and the efficacy of BMSC transplantation therapy in a rat POP model. We first identified FBLN5 as a target of miR-138. miR-138, fibulin-5 (FBLN5), interleukin-1β (IL-1β), and elastin expression in uterosacral ligament of POP patients and controls were detected by reverse transcription quantitative polymerase chain reaction (RT-qPCR) and western blot analysis. After isolation and identification, BMSCs were treated to alter their expression of miR-138 or FBLN5. Proliferation of BMSCs was analyzed by CCK-8. After establishing the rat pelvic floor dysfunction (PFD) model, we evaluated efficacy of BMSC injection by applying leak point pressure (LPP) and the conscious cystometry (CMG) tests. miR-138 inhibition resulted in increased viability of BMSCs and elevated their secretion of elastin, while downregulating IL-1β expression. BMSCs with inhibited miR-138 improved LPP and conscious CMG results *in vivo*. Taken together, miR-138 could be a potential therapeutic target for treating POP in conjunction with tissue engineering.

## INTRODUCTION

Pelvic floor dysfunction (PFD) refers to a group of clinical conditions, which include stress urinary incontinence, pelvic organ prolapse (POP), overactive bladder syndrome, and fecal incontinence [1]. POP entails the descent of one or more of the female pelvic organs (uterus, bladder, rectum) into the vagina [2]. Defined clinically by those symptoms, POP has prevalence of 3 - 6 %, and up to 50% based upon vaginal examination [3]. Among the main risk factors for POP are parity, vaginal delivery, age, and body mass index (BMI), and the preoperative stage is substantiated as a risk factor for POP recurrence [4]. Although severe morbidity from POP is unusual, surgical treatment may be provided for those in need of medical care if conservative treatments prove to be ineffective [5]. Moreover, the surgery has a lamentably high failure rate, which is associated with the poor quality of pelvic supportive tissues. Therefore, the identification of molecular pathways imparting and improved integrity of pelvic supportive tissues is critical for developing more successful treatment strategies for POP [6].

Tissue engineering uses a combination of cells, biomaterials, growth factors, and/or drugs implanted into an area of tissue damage or loss to accelerate repair and promote regeneration of the damaged or lost tissue [7]. Recent tissue engineering research has developed a new synthetic mesh with improved biocompatibility due to delivery of endometrial mesenchymal stem cells (eMSC) that can restore strength to prolapsed vaginal tissue in POP [8]. Bone marrow-derived mesenchymal cells (BMSCs) are among the best-characterized stem cells, which possess tremendous differentiation ability and the capacity to secrete factors beneficial for tissue repair [9]. Interestingly, a recent study combining microRNA (miRNA) biology, genetically engineered BMSCs, and nanoparticle technology, reported excellent results in treating PDF through delivery of miR-29, resulting in repression of elastin expression [9]. Other research showed that BMSCs could deliver microRNA-138-5p (miR-138-5p) to the astrocytes via exosomes, thus alleviating neuron injury in mice with ischemic stroke [10]. Furthermore, miR-138 can inhibit the differentiation of MSCs and thus modulate the treatment of POP by MSC regulation [11]. miRNA profiling from various patient cohorts has identified that miR-138 is involved in human diseases by directly binding to mRNAs, to alter their stability and transcription [12].

Vaginal delivery with obstetrical trauma is a risk factor for developing POP later in life. Loss of fibulin-5 (FBLN5), an elastogenesis-promoting cellular matrix protein, results in prolapse in mice [13], and seems likely to be involved in human POP. Indeed, the expression of FBLN5/Fibulin-5 is low in patients with POP [14]. FBLN5 modulates endothelial cell adhesion, cell growth, and motility, and is crucial for elastic fiber formation [15]. FBLN5 and elastin are mainly expressed in lamina propria and fibromuscular layers of the vagina and uterosacral ligament, but the expression of FBLN5 in uterosacral ligaments was enhanced in women with severe POP resulting in cervical elongation [16]. FBLN5/Fibulin-5 has been reported to inhibit the expression of interleukin-1β (IL-1β) [17], whereas other research indicates that IL-1β decreases elastin mRNA expression by inhibiting its transcription in lung interstitial fibroblasts [18]. Notably, local IL-1β might participate in the formation of aortic dissecting aneurysms by promoting extracellular matrix (ECM) metalloproteinase (MMP)-2 and MMP-9 and the breakage of elastin fibers, thus weakening the biomechanical properties of the aortic wall [19]. Furthermore, BMSCs have been established to significantly attenuate aortic elastin degradation and aortic aneurysm development as well as reduce IL-1β [20]. Related research showed that local oestrogen therapy (LET) regulated elastin expression to limit ECM degradation, which favor the integrity of the vaginal wall in post-menopausal women with severe POP [21]. Based on existing evidence, we hypothesized that miR-138 targets FBLN5 through IL-1β/elastin in BMSCs, thus presenting potential strategy to treat POP. To test this hypothesis, we examined miR-138 and FBLN5 expression in the uterosacral ligaments from women diagnosed with POP to provide a theoretical basis for developing targeted treatments.

## RESULTS

### BMSCs are successfully isolated from rats

**Figure 1:**
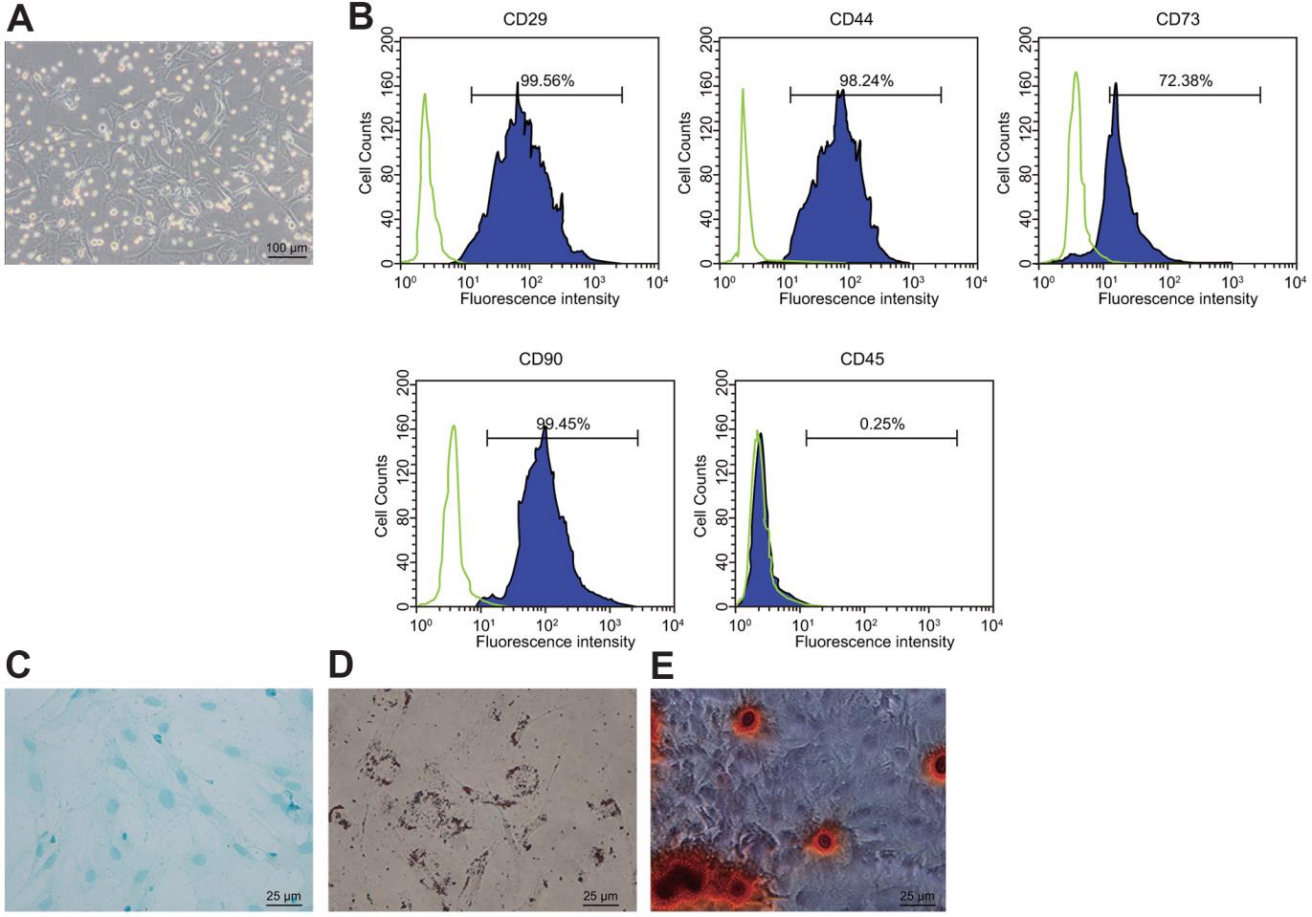
**Isolation and identification of BMSCs.** (**A**) condition of primary BMSCs after adherent growth for 7 d (100 ×); (**B**) expression of surface markers CD29, CD44, CD73, CD90, and CD45 in the BMSCs detected by flow cytometry; (**C**) Oil red O staining for the BMSCs after adipogenic induction (400 ×); (**D**) alizarin red staining for the BDMSCs after osteogenic differentiation (400 ×); (**E**) Alcian blue staining for BMSCs after chondrocyte induction (400 ×).

There are two stem cells in the bone marrow: MSCs and hematopoietic stem cells. BMSCs were isolated by the whole bone marrow adherence method after being seeded to the culture flask. After 7 d in culture, a large number of primary cells were seen, with the adherent cells showing an elongated shape under the microscope (Figure 1A). Flow cytometry revealed positive expression of CD44, CD73, and CD90 in the isolated MSCs and negative expression of hematopoietic cell marker CD45, indicating that the isolated cells were BMSCs (Figure 1B). After 14 d of osteogenic and adipogenic inductions, results confirmed that the isolated MSCs had bi-directional potential of adipogenic and osteogenic differentiation (Figure 1C, 1D). After 3 weeks of induction of the BMSCs in the chondroblast culture medium, the cells became plumper and more spindle-shaped than before the induction, which was the characteristic of differentiation to chondrocytes (Figure 1E).

### miR-138 targets FBLN5 in BMSCs

**Figure 2:**
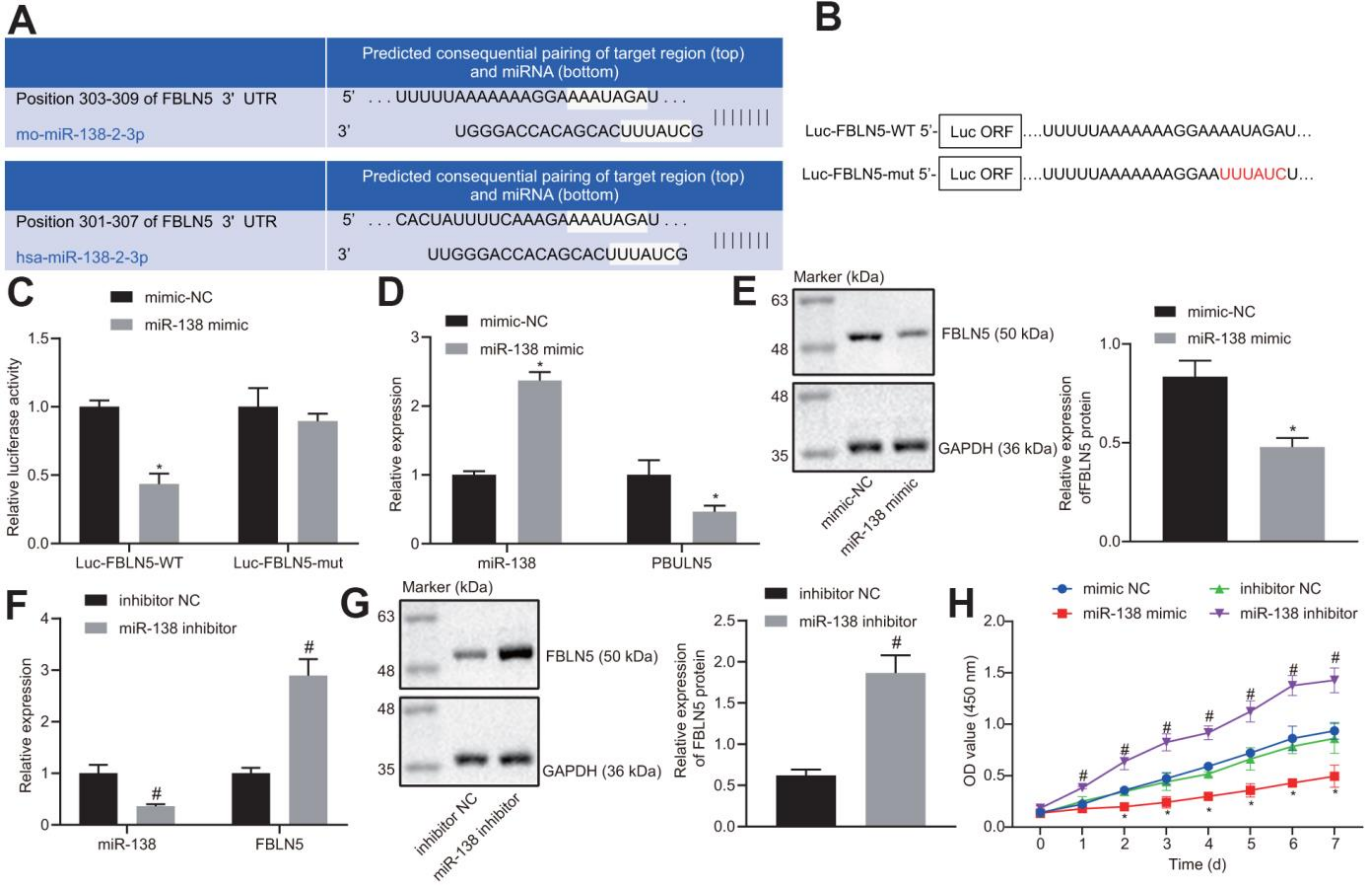
**The target relationship between FBLN5 and miR-138 is verified.** (**A**) sequence alignment of miR-138 with potential targeting site in the 3’-UTR of FBLN5 mRNA; (**B**) Wild-type (Luc-FBLN5-WT) or mutated (Luc-FBLN5-mut) targeting sequences from FBLN5 mRNA 3’-UTR were fused at the 3’ of the luciferase reporter open reading frame (Luc ORF); (**C**) Luciferase activities of Luc-FBLN5-WT and Luc-FBLN5-mut constructs were measured in BMSCs transfected with mimic NC or miR-138 mimic; (**D**) miR-138 expression and the mRNA expression of FBLN5 examined in BMSCs transfected with mimic NC or miR-138 mimic by RT-qPCR; (**E**) the protein expression of FBLN5 examined in BMSCs transfected with mimic NC or miR-138 mimic by western blot analysis; (**F**) miR-138 expression and the mRNA expression of FBLN5 examined in BMSCs transfected with inhibitor NC or miR-138 inhibitor by RT-qPCR; (**G**) the protein expression of FBLN5 examined in BMSCs transfected with inhibitor NC or miR-138 inhibitor by western blot analysis; (**H**) the OD value of BMSCs at the 0-7 d assessed by CCK-8. ^*^ *p* < 0.05 *vs*. BMSCs treated with mimic NC; ^#^ *p* < 0.05 *vs*. BMSCs treated with inhibitor NC; statistical data were measurement data, and described as mean ± standard deviation. The unpaired *t* test was conducted for comparison between two groups. The repeated measures ANOVA was applied for the comparison of data at different time points, followed by Bonferroni’s correction. The experiment was repeated 3 times independently.

TargetScan software revealed the binding sites between miR-138 and FBLN5 3’-UTR (Figure 2A). To validate whether miR-138 regulated FBLN5-3’-UTR through the binding sites, the wild-type and mutant type of FBLN5-3’-UTR were constructed and cloned into the downstream of a luciferase open reading frame (Figure 2B). At 24 h after these constructs were transfected together with miR-138 mimic into HEK-293T cells, the activity of the luciferase fused with FBLN5-wt targeting sequence was significantly reduced, whereas the FBLN5-mut was unaffected (Figure 2C), implying that this site on the 3’-UTR of FBLN5 mRNA was directly targeted by miR-138. The mRNA and protein expression of endogenous FBLN5 in BMSCs was significantly inhibited after the transfection of miR-1313 mimic (Figure 2D, 2E). The above results suggested that miR-138 was a negative regulator of FBLN5 in BMSCs. Next, miR-138 inhibitor was stably introduced into the cultured BMSCs. The expression of FBLN5 mRNA and protein in BMSCs transfected with miR-138 inhibitor was significantly increased compared to BMSCs transfected with inhibitor NC (Figure 2F, 2G). Moreover, CCK-8 assay showed the proliferation of BMSCs was inhibited by the transfection of miR-138 mimic relative to the mimic NC treatment, and the proliferation of BMSCs was improved by the transfection of miR-138 inhibitor compared to inhibitor NC transfection (Figure 2H). Thus, miR-138 could target FBLN5 in BMSCs, and the silencing of miR-138 promoted the proliferation of BMSCs.

### miR-138 inhibits the expression of elastin through the FBLN5/IL-1β axis

**Figure 3:**
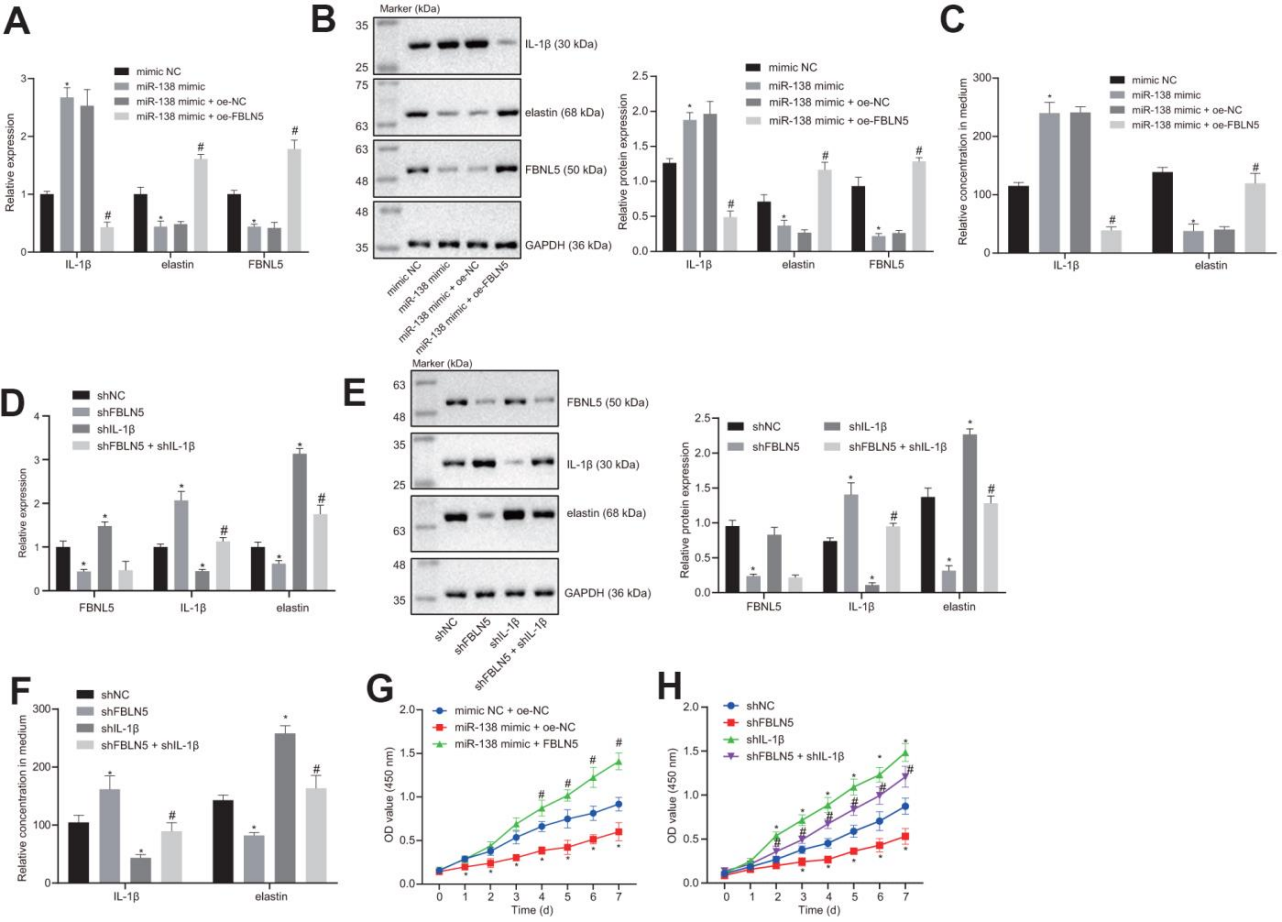
**miR-138 attenuates the proliferation of BMSCs by suppressing elastin and FBNL5 expression and potentiating IL-1β expression.** (**A**) the mRNA expression of FBNL5, elastin, and IL-1β in BMSCs treated with miR-138 mimic alone or in the presence of oe-FBNL5 measured by RT-qPCR; (**B**) the protein expression of FBNL5, elastin, and IL-1β in BMSCs treated with miR-138 mimic alone or in the presence of oe-FBNL5 measured by western blot analysis; (**C**) the release of elastin and IL-1β in BMSCs treated with miR-138 mimic alone or in the presence of oe-FBNL5 measured by ELISA; (**D**) the mRNA expression of FBNL5, elastin and IL-1β in BMSCs treated with sh-FBNL5 and/or sh-IL-1β measured by RT-qPCR; (**E**) the protein expression of FBNL5, elastin, and IL-1β in BMSCs treated with sh-FBNL5 and/or sh-IL-1β measured by western blot analysis; (**F**) the release of elastin and IL-1β in BMSCs treated with sh-FBNL5 and/or sh-IL-1β measured by ELISA; (**G**) the OD value of BMSCs treated with miR-138 mimic alone or in the presence of oe-FBNL5 at the 0-7 d assessed by CCK-8; (**H**) the OD value of BMSCs treated with sh-FBNL5 and/or sh-IL-1β at the 0-7 d assessed by CCK-8. ^*^ *p* < 0.05 *vs*. BMSCs treated with mimic NC, sh-NC or mimic-NC + oe-NC; ^#^ *p* < 0.05 *vs*. BMSCs treated with miR-138 mimic + oe-NC or sh-FBLN5; statistical data were measurement data, and described as mean ± standard deviation. The unpaired *t*-test was conducted for comparison between two groups. The one-way ANOVA was adopted for comparison among multiple groups, followed by Tukey’s *post hoc* test. The repeated measures ANOVA was applied for the comparison of data at different time points, followed by Bonferroni’s correction. The experiment was repeated 3 times independently.

The RT-qPCR and western blot results showed that the expression of IL-1β was increased, and the expression levels of elastin and FBNL5 were reduced following miR-138 mimic treatment relative to mimic NC. However, co-transfection of FBNL5 with miR-138 mimic reversed the effects of miR-138 on the expression of these genes (Figure 3A, 3B). ELISA assay also showed that miR-138 mimic transfection led to upregulation of IL-1β and downregulation of elastin, whereas the overexpression of FBNL5 rescued from these changes (Figure 3C). These results suggested that miR-138 could affect the expression of IL-1β and elastin through FBLN5. Next, we treated BMSCs with sh-FBLN5 or sh-IL-1β, which showed that, compared with the sh-NC treatment, the expression of the FBLN5 and elastin was significantly reduced following the sh-FBLN5 treatment, whereas the expression of IL-1β was significantly increased. However, the expression of the elastin was significantly increased, and the expression of IL-1β was significantly decreased by sh-IL-1β treatment. Compared with sh-FBLN5 single transfection, the simultaneous silencing of FBLN5 and IL-1β provoked an enhancement in elastin expression, but a downregulation in IL-1β expression (Figure 3D–3F).

The results of CCK-8 showed that the proliferation of BMSCs after miR-138 mimic + oe-NC delivery was inhibited compared with mimic NC + oe-NC, and the proliferation of BMSCs following miR-138 mimic + oe-FBLN5 was promoted compared with miR-138 mimic + oe-NC (Figure 3G). sh-FBLN5 exhibited an anti-proliferation action, while sh-IL-1β exhibited a pro-proliferation action relative to sh-NC. Nevertheless, the combination of sh-FBLN5 and sh-IL-1β stimulated the proliferation compared with sh-FBLN5 treatment alone (Figure 3H). In summary, miR-138 affects the proliferation of BMSCs by inhibiting elastin expression via the FBLN5/IL-1β axis.

### The BMSCs containing miR-138 antagomir alleviates POP in the PFD rats

**Figure 4:**
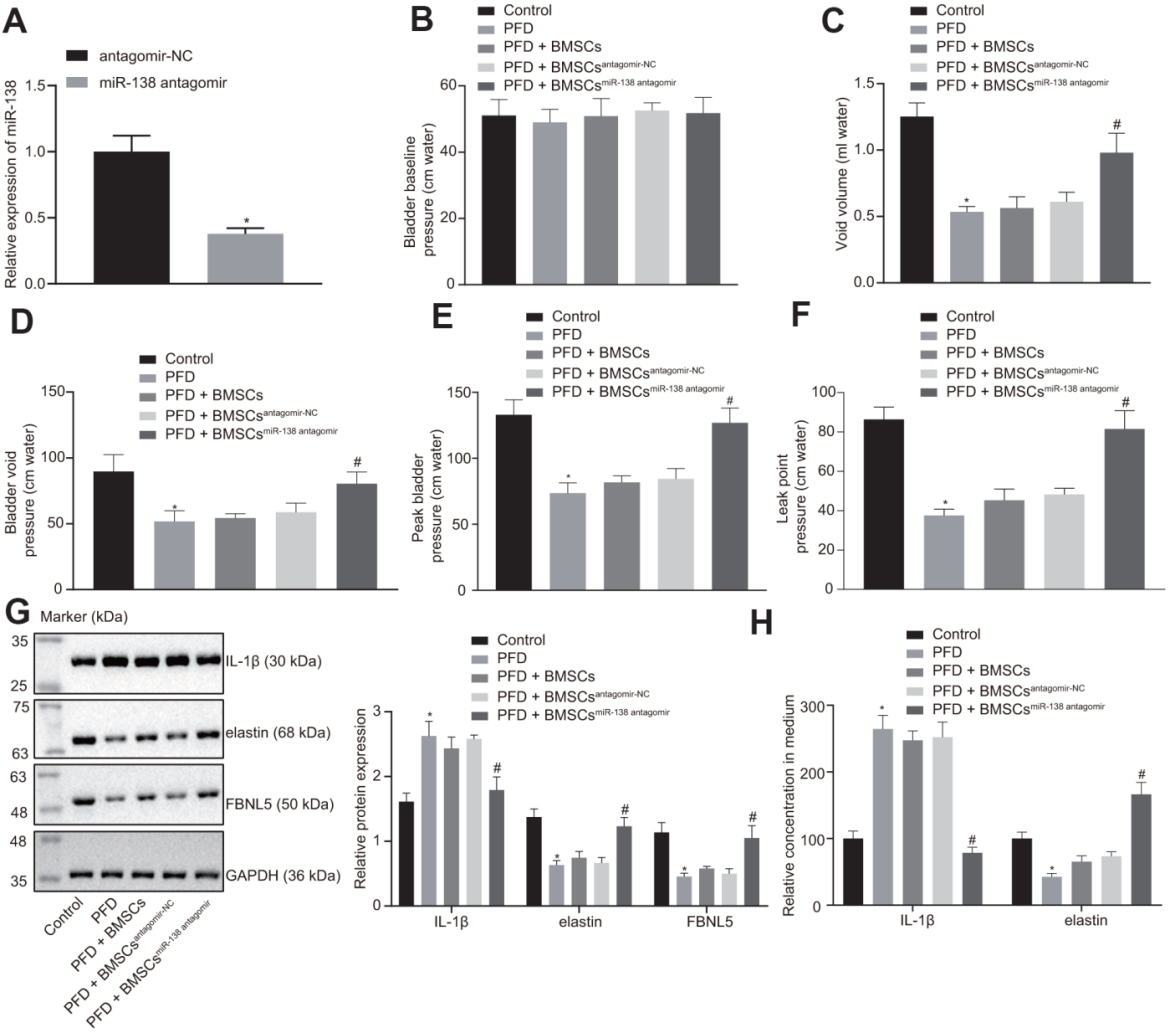
**miR-138 inhibition improves results of urodynamic tests in PFD rats after BMSC transplant.** (**A**) miR-138 expression in BMSCs treated with antagomir-NC or miR-138 antagomir evaluated by RT-qPCR; (**B**) basal bladder pressure level of rats in each group; (**C**) bladder void volume of rats in each group; (**D**) bladder void pressure of rats in each group; (**E**) peak bladder pressure of rats in each group; (**F**) LPP of rats in each group; (**G**) the protein expression of IL-1β, elastin, and FBNL5 in rats evaluated by western blot analysis; (**H**) the content of IL-1β and elastin in peripheral blood of rats examined by ELISA. ^*^ *p* < 0.05 *vs*. BMSCs treated with antagomir-NC or rats injected with saline (control rats); ^#^ *p* < 0.05 *vs*. PFD rats injected with BMSCs^antagomir-NC^; statistical data were measurement data, and described as mean ± standard deviation. The unpaired *t* test was conducted for comparison between two groups. The one-way ANOVA was adopted for comparison among multiple groups, followed by Tukey’s *post hoc* test. The experiment was repeated 3 times independently.

We next established a rat PFD model to test the effect of miR-138-inhibited BMSC delivery on PFD symptoms *in vivo*. We first treated BMSCs with antagomir-NC or miR-138 antagomir and detected the expression of miR-138 in BMSCs by RT-qPCR, which showed reduced expression of miR-138 in BMSCs treated with miR-138 antagomir compared with antagomir-NC group, as shown in Figure 4A. Next, we observed similar baseline bladder pressure levels among all rats in conscious CMG tests (Figure 4B). In the PFD rats, the void volume and bladder void pressure were significantly diminished compared to the control rats (Figure 4C, 4D). Also, the peak bladder pressure and LPP in PFD rats were significantly lower than in control rats (Figure 4E). All the above results indicated the successful establishment of the PFD model. Next, found that rats treated with injections of BMSCs or BMSCs transfected with antagomir-NC exhibited slightly increased void volume and bladder void pressure. Importantly, rats receiving injections of miR-138 antagomir transfected BMSCs showed the decreased void volume and enhanced bladder void pressure. As expected, injection of control BMSCs exhibited almost no effect on peak bladder and LPP relative to control PFD rats with saline treatment, while rats with injection of miR-138 antagomir transfected BMSCs showed significantly increased void volume and LPP (Figure 4E, 4F).

Western blot analysis showed attenuation of elastin and FBNL5 levels while promotion of IL-1β levels in PFD rats compared to the control rats. The rats with injection of BMSCs and injection of BMSCs transfected with antagomir-NC showed slightly reduced expression of IL-1β, and increased expression of elastin and FBNL5, whereas injection of miR-138 antagomir transfected BMSCs showed significant trends (Figure 4G). These results suggested that treatment with miR-138-inhibited BMSCs significantly alleviated POP symptoms in PFD rats.

### Comparison of basic data from eligible selected subjects

As listed in Table 1, the 94 subjects enrolled in this study were divided into that POP group (46 POP patients received pelvic surgery or other treatments) and non-POP control group (48 subjects receiving abdominal hysterectomy for uterine leiomyoma or cervical cancer). The age distribution and BMI showed no significant group differences. Although the parity times in the POP group were higher than the non-POP group, the difference was not significant. There were 37 menopausal and 9 non-menopausal women in the POP group, and 35 menopausal and 13 post-menopausal women in the non-POP group, respectively. Thus, there was no difference in menopause status between the two groups. There was no significant group difference for family history of POP, with 5 women in the POP group and 3 cases in the non-POP group showing a previous family history. Based on the POP-Q score results, the 46 POP patients were divided into the POP II group (n = 24) and the POP III group (n = 22), which did not differ with respect to age, BMI, parity, menopause status, and family history.

**Table 1:** Comparison of basic data from eligible selected POP patients and non-POP controls.

	**Age (year)**	**BMI (kg/m^2^)**	**Parity (times)**	**Menopause status**	**Family history of POP**
Non-POP group (n = 48)	53.31 ± 10.65	21.49 ± 2.31	2.34 ± 0.87	35/48	3/48
POP group (n = 46)	55.46 ± 8.59	24.47 ± 2.73	2.63 ± 0.97	37/46	5/46
POP II (n = 24)	54.23 ± 12.51	24.12 ± 1.78	2.79 ± 1.54	17/24	3/24
POP III (n = 22)	57.85 ± 11.39	25.38 ± 2.52	2.95 ± 1.34	20/22	2/22

Note: Continuous variable was examined by independent samples *t*-test and its results were shown by mean ± standard deviation. Based on the POP-Q score results, all patients were identified as POP-Q score > stage II. BMI, body mass index; POP, pelvic organ prolapse; POP-Q, pelvic organ prolapse quantification.

**Figure 5:**
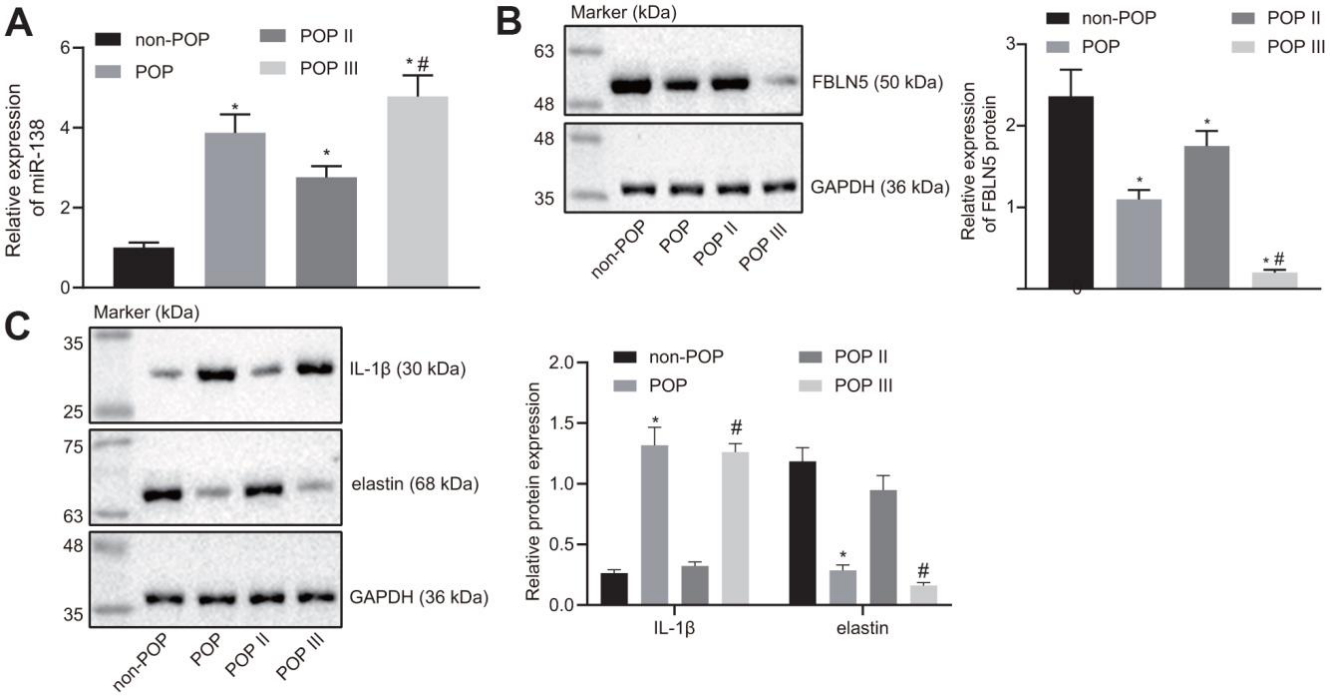
**miR-138 and IL-1β are overexpressed and FBLN5 and elastin are repressed in POP patients.** (**A**) miR-138 expression in patients with POP or non-POP controls measured by RT-qPCR, N = 46; (**B**) the protein expression of FBLN5 in patients with POP or non-POP controls measured by western blot analysis; (**C**) the protein expression of IL-1β and elastin in patients with POP or non-POP controls measured by western blot analysis. ^*^ *p* < 0.05 *vs*. non-POP controls; ^#^ *p* < 0.05 *vs*. POP II patients; statistical data were measurement data and calculated as mean ± standard deviation. The one-way ANOVA was adopted for comparison among multiple groups, followed by Tukey’s *post hoc* test.

The comparison of results of RT-qPCR results for miR-138 expression between groups are shown in Figure 5A. Notably, the miR-138 expression was significantly higher in the POP group than the non-POP group, whereas miR-138 expression was significantly higher in the POP III group than in the POP II group, suggesting that miR-138 expression might correlate with POP severity.

As shown in Figure 5B, western blot results showed that the expression of FBLN5 was lower in the POP group compared to the non-POP group. Further, FBLN5 expression was also significantly different between the POP III and POP II groups, where the POP II group showed remarkably higher FBLN5 expression compared to the POP III group, this suggesting that FBLN5 expression could also predict the severity of POP.

In addition, western blot analysis showed that the expression of elastin was lower in the POP group than in the non-POP group, while IL-1β expression was higher in the POP group than the non-POP group, and differences of greater significance were seen in the POP III group (Figure 5C), indicating that miR-138 may inhibit the expression of elastin through the FBLN5/IL-1β axis and affect POP progression.

## DISCUSSION

The occurrence of PFD is closely linked to elasticity, toughness, and functional changes of the connective tissues of the pelvic support tissues, and due to its accessibility, the transplantation of BMSCs may be applicable in the context of ligament tissue engineering [22]. Therefore, our study explored the effect of miR-138 modified BMSCs on the recovery of POP. The findings of our study revealed that miR-138 targeted FBLN5 to upregulate IL-1β expression and downregulate elastin expression, thus inhibiting proliferation of BMSCs and alleviating POP progress.

We found that the rat femur BMSCs exhibit positive expression of CD44, CD73, and CD90, but not CD45, and that these cells could be induced into osteogenic cells and adipogenic cells, indicating that our primary cultured cells have the expected characteristics of BMSCs. MSC injection has the capacity to improve bladder function in a previous study investigating a rat model of bladder outlet obstruction [23]. Moreover, anti-miR-138 delivery down-regulated the endogenous miR-138 expression in BMSC sheets, whereas corresponding *in vivo* results revealed a robust stimulating effect of the delivery of anti-miR-138 delivery on the bone regeneration ability of BMSC sheets [24]. Consistent with that result, the present study uncovered that silencing of miR-138 promoted the proliferation of BMSCs. Moreover, we found that miR-138 expression was increased in BMSCs and clinical samples of POP patients. In with line our present data, women diagnosed with grade III POP have been observed in an earlier study to exhibit elevated miR-92 expression in uterosacral ligaments, and showed significantly lower estrogen receptor β1 expression in the same tissues, when compared with patients with grade II POP [25]. Also, the mean expression of miR-221/222 was enhanced by approximately twofold in women with POP relative to controls [26]. In that study, Shi et al. confirmed that un-stimulated MSCs are incapable of immunosuppression, but that upon stimulation with the supernatant of activated lymphocytes, or with combinations of IFN-γ with IL-1β, the MSCs became potently immunosuppressive, making them more suitable for application in treating tissue injuries induced by immune responses [27]. To conclude, miR-138 silencing combined with BMSC injection might further repair tissue structures and tighten connective tissues. To uncover the underlying mechanism of miR-138 in POP, we searched for target genes through bioinformatic analyses (http://www.targetscan.org/) and dual luciferase reporter gene assay. Subsequently, we found that FBLN5 expression was decreased in BMSCs and clinical samples of POP patients. Further, FBLN5 emerged as a putative target for miR-138 via regulation at the post-transcriptional level. Vaginal tissue FBLN5 is of great importance for baseline pelvic organ support and for protection and recovery from parturition- and elastase-stimulated prolapse [28]. In women from the Russian Federation, several common SNPs of the FBLN5 were correlated with advanced POP, especially as occurred after pelvic floor injury [29]. Moreover, leiomyomas had low expression of FBLN5, which validated as a direct target of miR-200c through interaction with its 3’UTR [30].

This study also illustrated that miR-138 downregulation could enhance the expression of elastin and FBLN5, while reducing the expression of IL-1β. Similarly, a previous study provided strong evidence that miR-214 could promote fibroblast differentiation of adipose-derived MSCs by restoring elastin expression to improve PFD in rats with birth trauma [31]. Interestingly, FBLN5 plays dual critical roles in elastic fiber homeostasis in the extracellular matrix, on the one hand by promoting the generation of elastic fibers and on the other hand by repressing matrix degradation [32]. Previously, treatment of fibroblasts with IL-1β abolished FBLN5 mRNA expression, and the coordinate expression of FBLN5 with elastin in lung fibroblasts seemed to play a key role during lung injury and repair [18]. Elastin, an important factor of vaginal and pelvic floor connective tissues, is exposed to extraordinary forces and mechanical stretching in the process of childbirth [29]. The severity of POP and gene expression of ECM-related proteins Fibulin-5 and elastin were found to be inversely correlated in vaginal tissues from normal and elongated cervices [16]. Further, a recently published study showed that miR-29a-3p inhibition resulted in upregulated expression and secretion of elastin in culture of BMSCs *in vitro* in a PFD model [9]. Our rescue experiments displayed that silencing of IL-1β in the presence of downregulation of FBLN5 promoted elastin expression, while diminished IL-1β expression in BMSCs.

**Figure 6:**
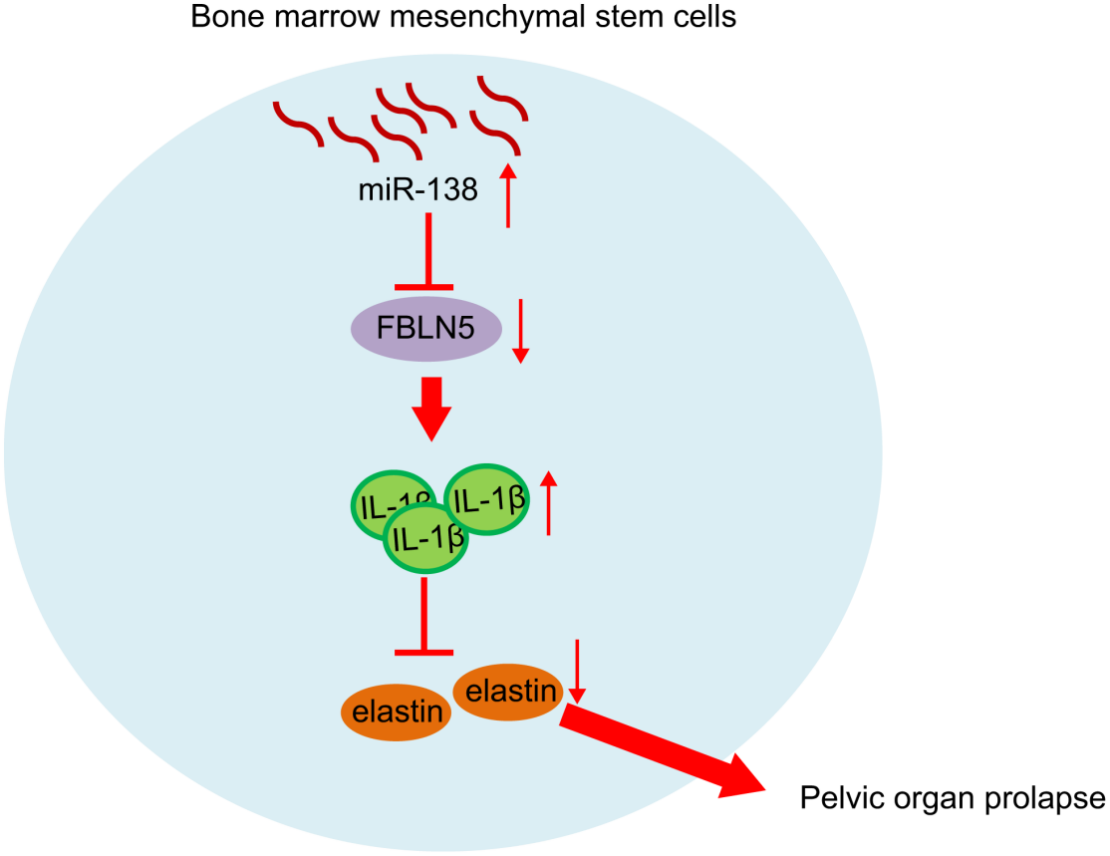
**miR-138 promoted the expression of IL-1β by targeting FBLN5, and the up-regulation of IL-1β expression inhibited the expression of elastin and promoted POP progression.** After silencing miR-138 in BMSCs and transplant, the POP in PFD rats was alleviated.

In conclusion, we demonstrated that miR-138 was upregulated in BMSCs from rats with PFD and in patients with POP, and that inhibition of miR-138 in BMSCs inhibited cell proliferation by targeting FBLN5 through the IL-1β/elastin axis (Figure 6). Our findings provide a novel insight that miR-138 inhibition via BMSCs delivery may provide a novel therapeutic approach for treatment of POP. However, the specific mechanism of POP treatment is not yet known, and further empirical studies are needed.

## MATERIALS AND METHODS

### Ethics statement

All experiments were performed following the approval of the review board for human research of the Third Affiliated Hospital of Zhengzhou University, and informed consent was obtained from all of the participating women (Human Research Ethics Approval: #201706003). The animal study was carried out in strict accordance with the recommendations in the Guide for the Care and Use of Laboratory Animals of the National Institutes of Health, and all efforts were taken to minimize discomfort of the experimental animals. The protocol was approved by the Committee on the Ethics of Animal Experiments of the Third Affiliated Hospital of Zhengzhou University (Animal Research Ethics Approval: #201903002).

### Tissue collection

From July 2017 to September 2019, 46 patients with POP who underwent vaginal hysterectomy or other treatments in the Third Affiliated Hospital of Zhengzhou University were enrolled in this study. The enrolled patients were aged more than 45 years, and had had at least one delivery. Diagnosis of POP was confirmed by prolapse of the uterus and the vaginal vault with different degree of anterior or posterior vaginal wall prolapse to gynecological examination, with a score II in the POP-quantification (POP-Q) scale for rating the degree of prolapse. Any patients with clinical diagnosis of chronic pelvic inflammation, endometriosis, congenital connective tissue disease, or related pulmonary diseases (such as chronic obstructive pulmonary disease), and those who had undergone pelvic surgery, or received hormone treatment within three months prior to the study were excluded. Meanwhile, 48 non-POP control patients were recruited during the same period at the same hospital. These patients were also aged at least 45 years and had a POP-Q score < stage I to gynecological examination, and had a history of at least one delivery. These control patients were undergoing hysterectomy for uterine fibroids or cervical cancer. The controls were also free of chronic pelvic inflammatory disease, endometriosis, congenital connective tissue disease, related pulmonary diseases as well as pelvic surgery or hormone treatment. The clinical data included age, parity, medical family history, and fasting height and body weight were collected in a health questionnaire from all subjects, and the BMI was calculated as = weight/(height)^2^ (kg/m^2^).

Fresh uterosacral ligament tissues were collected from the junction of the ligament and cervix (0.5-1.0 cm) from all POP patients who underwent complete hysterectomy and likewise from the non-POP patients. Fresh tissue specimens were flash-frozen in liquid nitrogen and stored at -80° C for later use [25].

### Primary rat BMSC isolation and culture

Six-month-old female Sprague Dawley rats were utilized in the animal study. Rats were deeply anesthetized, and femurs were extracted to obtain bone marrow cells, which were flushed from the fractured femurs using 5-10 mL ice-cold Iscove’s modified Dulbecco medium (IMDM). The obtained cell suspension was centrifuged at 150 ×g for 5 min and the pelleted cells were resuspended in IMDM. The cell suspension was layered in Percol separation solution (density 1.073 g/mL, Sigma-Aldrich, St. Louis, MO, USA) and centrifuged at 400 ×g for 30 min at 26° C. The resulting cotton-like cells were collected at the interface and rinsed one time before being cultured with IMDM containing 20% fetal bovine serum (FBS) and 1% streptomycin/penicillin (Sigma-Aldrich). The medium was renewed after 24 h, and the cells were maintained in the same medium until reaching approximately 80% confluence. Cells were then trypsinized, replated, and cultured until passage two. The plastic-adhering fibroblast-like cells obtained from these two passages were regarded as MSCs, and were stored for use in subsequent experiments [1].

### Analysis of BMSC surface markers by flow cytometry

BMSCs were harvested and stained with phycoerythrin (PE)-conjugated rat antibody to CD44 (ab23396, Abcam, Cambridge, UK), PE-conjugated rat antibody to CD73 (#12-0731-82, Invitrogen Inc., Carlsbad, CA, USA), PE-conjugated rat antibody to CD90 (ab33694, Abcam, Cambridge, IK), fluorescein isothiocyanate-conjugated rat antibody to CD45 (ab33916, Abcam, Cambridge, UK), PE-conjugated CD29 (ab33916, Abcam, Cambridge, UK), and affinity-purified rat antibody to IgG1 as isotype control. Fluorescence-activated cell sorting was carried out with FACSDIva (Canto, BD Bioscience, San Jose, CA, USA), and the data was analyzed using the FlowJo software (Tree Star, Ashland, OR, USA) [11].

### Adipogenic and osteogenic differentiation of BMSCs

Adipogenic differentiation solution was prepared with high glucose Dulbecco’s modified Eagle’s medium (DMEM) containing 10% FBS, 1 μM dexamethasone, 0.5 mM isobutylmethyl xanthine, 200 μM indomethacin, and 10 μg/mL insulin (Sigma-Aldrich). The high glucose DMEM containing 10% FBS and 10 μg/mL insulin was used as adipose maintenance solution. The adipogenic induction was performed by cultivating cells in adipogenic differentiation solution for 3 d and then in adipose maintenance solution for 1 d. This process was repeated 3 times, and the cells were further cultured in adipogenic differentiation solution for 2 d. The adipogenic differentiation thus took 14 d. High glucose DMEM containing 10% FBS, 1 μM dexamethasone, 10 mM β-sodium glycerophosphate, and 50 mg/L vitamin C (Sigma-Aldrich) was utilized as osteogenic induction medium. The medium was renewed at 72 h intervals. The osteogenic differentiation also took 14 d.

Oil red O staining and alizarin red staining were used to identify adipogenic and osteogenic differentiation abilities of BMSCs after 14 d of differentiation. Cells were fixed in 4% paraformaldehyde at room temperature. After 15 min, the cells were stained with Oil red O or alizarin red (Sigma-Aldrich) for 1 h, washed in distilled water, and photographed with an inverted microscope.

The obtained 2.5 × 10^5^ MSCs was induced by 5 mL cartilage differentiation medium supplemented with 10 μg TGFβ1 (R&D System, Los Angeles, CA, USA), 50 μg insulin-like growth factor 1 (R&D System), and 2 mg/mL dexamethasone (Sigma-Aldrich). The medium was refreshed at 3 d intervals, and the cells was cultivated for 3 weeks and then stained with Alcian blue [31].

### Cell transfection

The miR-138 mimic and its negative control (NC) were purchased from Sigma-Aldrich, and were transfected into HEK-293T cells or BMSCs using Lipofectamine 2000 reagent (Invitrogen). miR-138 inhibitor and its NC were purchased from Sigma-Aldrich and packaged for transduction to create stable cell lines according to the manufacturer’s protocols. miR-138 expression was determined with a TaqMan Advanced miRNA Assay Kit (Applied Biosystems, Waltham, MA, USA) according to the manufacturer’s instructions [9].

The full-length sequence of CDS amplified by specific primers PCR with HindIII and XhoI cleavage sites was inserted into the expression vector pcDNA3.1 to construct FBLN5 overexpression vector pcDNA3.1-FBLN5. Antagomir were also transfected into BMSCs with Lipofectamine 2000 (Invitrogen). In brief, 20 pmol antagomir was added into 50 μL DMEM serum-free medium, and then 1 μL Lipofectamine was diluted into 50 μL serum-free DMEM, and the mixture was kept at room temperature for 5 min. Then, diluted antagomir and RNAi-Mate reagent were gently mixed and allowed to sit at room temperature for 20 min to form the antagomir/Lipofectamine complex. After standing, 100 μL antagomir/Lipofectamine complex was added into the corresponding cells, which were then incubated for 6 h at 37° C. After changing the medium once, cells experiments were carried out after further 48 h of culture. Antagomir was purchased from Guangzhou Kangchengjian Biotechnology Co., Ltd. (Guangdong, China) [33].

Sh-NC, sh-FBLN5, and sh-IL-1β (final concentration of 50 nM) were synthesized by Aksomics (Shanghai, China) [34, 35].

### Reverse transcription quantitative polymerase chain reaction (RT-qPCR)

Total RNA was isolated from cells with a RNeasy Plus Mini Kit (Qiagen, Gathersburg, MD, USA) following the manufacturer’s instructions. Reverse transcription was performed using a PrimeScript RT reagent Kit (Promega, Madison, WI, USA). The gene expression was quantified by SYBR Green Master Mix (Life Technologies, Carlsbad, CA, USA) after the reverse transcription. The cDNA obtained by reverse transcription was subjected to the Mir-XTM miRNA First Strand Synthesis Kit (Takara, Dalian, Liaoning, China) for the detection of miRNAs. Quantification of miR-138 expression was carried out by a Mir-XTM miRNA RT-qPCR TB Green^TM^ Kit (Takara). The primer sequences are shown in Table 2. The 2^-ΔΔCt^ method was used to calculate the relative expression of target genes and miRNA. mRNA expression was normalized to glyceraldehyde-3-phosphate dehydrogenase (GAPDH) expression, and expression of miR-138 was normalized to U6.

**Table 2:** Primer sequences for RT-qPCR.

	**Forward (5’-3’)**	**Reverse (5’-3’)**
miR-138	GCCGCAGCTGGTGTTGT GAAT	GCGAGCACAGAATTAATACGAC
GAPDH	ACCACAGTCCATGCCATCAC	TCCACCACCCTGTTGCTGT
IL-1β	GACTTCACCATGGAACCCGT	GGAGACTGCCCATTCTCGAC
U6	CTCGCTTCGG CAGCACA	GCGAGCACAGAATTAATACGAC
FBLN5	ACTGAAGGGGGTTAAGCGAAA	GAGGGATGCACAGATACCCG
elastin	GCCATTCCTGGTGGAGTTCCTGGA	ACCGCACCTGCAGACACTCCTAAG

Note: RT-qPCR, reverse transcription quantitative polymerase chain reaction; miR-138, microRNA-138; GAPDH, glyceraldehyde-3-phosphate dehydrogenase; IL-1β, interleukin-1β; FBLN5, fibulin-5.

### Western blot

Tissues or cells were thoroughly ground and lysed in a pre-cooled glass grinder containing pre-cooled lysis buffer composed of phenylmethanesulfonyl fluoride and phosphatase inhibitors. Next, 5× protein loading buffer was added to the protein sample. The protein was separated by sodium dodecyl sulfate-polyacrylamide gel electrophoresis and transferred to polyvinylidene difluoride membranes (Millipore Corp, Billerica, MA, USA). The membranes were blocked with 5% bovine serum albumin at room temperature for 1 h and then incubated with primary rabbit antibodies to FBLN5 (1:500, ab202977), IL-1β (1:1000, ab9722), elastin (1:500, ab217356), and GAPDH (1:10000, ab181602) overnight at 4° C. Then the membranes were incubated with secondary goat anti-rabbit antibody horseradish peroxidase-conjugated antibody to IgG H&L (1:5000, ab205718) at room temperature for 1 h and visualized with enhanced chemiluminescence method. All antibodies were purchased from Abcam. Data were analyzed with the Molecular Imager (Gel DocTM XR, 170-8170) and the associated software Quantity One-4.6.5 (Bio-Rad Laboratories, Hercules, CA, USA).

### Cell counting kit-8 (CCK-8) assay

For assessing proliferation, a CCK-8 assay was performed. BMSCs were seeded in 96-well plates at a density of 10,000 cells/well. Then, CCK-8 reagent (Dojindo Laboratories, Kumamoto, Japan) was added to each well and incubated for 1 h. The optical density (OD) value of the formazan product was detected at 450 nm with a 96-well spectrophotometric plate reader.

### Enzyme-linked immunosorbent assay (ELISA)

The expression of elastin and IL-1β in culture medium and peripheral blood of rats was detected by ELISA Kits (ml037153, Shanghai Enzyme-linked Biotechnology Co., Ltd., Shanghai, China) and (ml059373, Shanghai Enzyme-linked Biotechnology Co., Ltd.), respectively, according to the manufacturer's instructions.

### Dual luciferase reporter gene assay

According to the prediction results of TargetScan (http://www.targetscan.org/) software, the 3’-untranslated region (UTR) of FBLN5 that contained the miR-214 target sequence was cloned into the luciferase reporter vector pGL4 (Promega Corporation, Madison, WI, USA) for the construction of the pGL4-FBLN5-wt-3’-UTR plasmid. The core sequence of the 3’ UTR of FBLN5 (the target gene of miR-138) was mutated to construct the luciferase reporter plasmid PGL4-FBLN5-mut-3’-UTR (used as the control) containing FBLN5 3’-UTR mutant. The previously constructed luciferase reporter plasmid and mutant plasmid were co-transfected with the miR-138 mimic or mimic NC into HEK-293T cells. Luciferase activity was detected via a dual-luciferase reporter assay (Promega, Madison, WI, USA) after 24 h of transfection.

### Induction of a rat PFD model

Six month old female SD rats were randomly divided into 5 groups of 12 animals each: (1) the healthy control group, which were normal rats without any intervention during the whole experimental period; (2) the PFD group, which received saline injection into the weakest part of pelvis 14 d after vaginal dilation to induce PFD symptoms; (3) the PFD + BMSCs group, in which BMSCs were administered at 14 days after establishment of the PFD model; (4) PFD + BMSCs ^antagomir-NC^ group, which were treated with BMSCs transfected with antagomir-NC at 14 days after establishment of the PFD model; and (5) the PFD + BMSCs ^miR-138 antagomir^ group, which received BMSCs transfected with miR-138 antagomir at 14 d after the operation to induce PDF. Here, 500 μL of a solution containing 8 × 10^5^ BMSCs or modified BMSCs were injected into via tail [36–38]. On the 7th day after the various treatments, conscious cystometry (CMG) and leak point pressure (LPP) urodynamic tests were carried out.

The specific steps for construction of the PFD rat model are as follows: an 18F catheter was inserted into the rat vagina and then fixed with a single 3-0 silk suture. The Foley balloon was inflated with 2.5-3.0 mL water and connected to a pressure transducer (about 0.15 kg) to exert pressure on the pelvic floor support tissues. After 4 h, the catheter was deflated and removed along with the vaginal pressure transducer. 14 d after vaginal dilatation, CMG and LPP tests were measured to ensure the successful establishment of PFD model and to evaluate the therapeutic effect of BMSCs injection.

### CMG test

Two d prior to the conscious CMG test, the bladder catheter (PE-50 tubing with a flitted tip) was inserted into the PFD rats. The catheter was connected to a syringe pump (KD Scientific, New Hope, PA, USA) and a pressure transducer (Grass Instruments, West Warwick, RI, USA). Each bladder was filled with saline solution through the catheter at a speed of 5 mL/h. A voiding contraction was defined as the increase in bladder pressure, which resulted in a loss of urine volume, as detected by a calibrated force transducer (Grass Instruments). Three filling and voidings were recorded in each rat. The mean values of bladder baseline pressure, urinary volume, peak voiding pressure, and urinary pressure increase (peak urination pressure minus the bladder baseline pressure) was calculated for each animal, according to chart recorder results [9].

### LPP test

Two d prior to the test, the bladder catheter (PE-50 tubing with a flitted tip) was inserted into the PFD rats and connected to the pressure transducer and the flow pump via a stopcock. Under ethyl carbamate anesthesia (at 1.2 g/kg intraperitoneally), the bladder was palpated empty and filled with saline at a flow rate of 5 mL/h. When 0.3 mL was attained (about half the bladder capacity of a 200 g rat), gentle pressure from one finger was applied on the rat’s abdomen to increase the bladder pressure while continuously recording. The pressure slowly increased until the rats leaked saline through the urethra. At the first sign of leakage at the urethral orifice, the externally applied abdominal pressure was removed. The peak pressure was recorded when there was no detrusor contraction. The LPP was calculated by subtracting the bladder baseline pressure from the bladder peak pressure. The bladder was drained and refilled, and the procedure was repeated three times in each rat. The mean bladder baseline pressure and mean LPP were calculated for each rat [9].

### Statistical analysis

Statistical analysis was performed with the SPSS software 21.0 (IBM Corporation, Armonk, NY, USA). Measurement data (continuous variables) were expressed as mean ± standard deviation. The statistical significance of unpaired data with normal distribution and equal variance was calculated using unpaired *t*-test. Differences between multi-groups were analyzed by one-way analysis of variance (ANOVA) with Tukey’s *post hoc* test. The repeated measures ANOVA was applied for the comparison of data at different time points, followed by Bonferroni’s correction. A value of *p* < 0.05 indicated statistical significance.
